# Histology of neurogenic heterotopic ossification and comparison with its radiological expression in acute spinal cord injured patients

**DOI:** 10.1038/s41393-025-01070-4

**Published:** 2025-03-13

**Authors:** Eugen Ulrich, Alexis Brinkemper, Manfred Köller, Ingo Stricker, Astrid Gisevius, Thomas A. Schildhauer, Renate Meindl, Dennis Grasmücke, Sabrina Buche-Lyding, Volkmar Nicolas, Mirko Aach

**Affiliations:** 1 Department of Spinal Cord Injuries, BG University Hospital Bergmannsheil, Ruhr-University Bochum, Bürkle-de-la-Camp-Platz 1, 44789 Bochum, Germany; 2 Department of General and Trauma Surgery, BG-University Hospital Bergmannsheil, Ruhr-University Bochum, Bürkle-de-la-Camp-Platz 1, 44789 Bochum, Germany; 3 Georgius Agricola Stiftung Ruhr, Institute of Pathology, Ruhr-University Bochum, Bürkle-de-la-Camp-Platz 1, 44789 Bochum, Germany; 4 Institute for Diagnostic Radiology, Interventional Radiology and Nuclear Medicine, BG University Hospital Bergmannsheil, Ruhr-University Bochum, Bürkle-de-la-Camp-Platz 1, 44789 Bochum, Germany

**Keywords:** Trauma, Signs and symptoms

## Abstract

**Study design:**

Clinical prospective study.

**Objectives:**

To histologically examine puncture biopsies of early neurogenic heterotopic ossification (NHO) in spinal cord injured individuals.

**Setting:**

University of Bochum, Germany.

**Methods:**

After acute spinal trauma, participants underwent a sonographic examination of the hip joints and a routine laboratory examination. Magnetic resonance imaging (MRI) or computed tomography (CT) of the pelvis was performed if there were clinical and laboratory signs of NHO and sonographic evidence of edema and/or calcifications in the tissue around the hip joint. If NHO were detected, tissue was obtained and preserved for histological examination from the involved tissue around the hip joint affected by NHO and from an unaffected calf as control. Nine participants with a complete spinal cord lesion American Spinal Injury Association Impairment Scale (AIS) grade A and evidence of an acute NHO in the hip joint muscles were recruited for the study.

**Results:**

In all sonographic examinations of the hip joint, edematous changes could be detected. In one case, calcifications were detected sonographically. In MRI/CT in six participants, ossification could already be detected. All histological specimens from the NHO-affected gluteal region showed varying degrees of tissue deformation. The unaffected reference samples showed regular muscular structure microscopically.

**Conclusions:**

It was possible to show and compare histological changes in NHO-affected tissue with MRI/CT imaging, depending on the stage of NHO.

**Trial registration:**

DRKS, DRKS00034049. Registered 12 April 2024 - Retrospectively registered, https://www.drks.de/DRKS00034049.

## Introduction

Heterotopic ossification (HO) is a common and feared complication after the onset of acute paraplegia. When it occurs after damage to the central nervous system, it is also referred to as neurogenic heterotopic ossification (NHO) in the literature [1–3]. Study-dependent, NHO occurs in up to 25% of individuals affected by acute paraplegia [2–4]. The high variance in incidence results from varying neurological level of injuries in the spinal cord and manifestations of neurologic deficits, with sensorimotor complete lesion being a particular risk factor [5, 6]. NHO results in formations of laminar bone tissue in the area of joint-surrounding muscles, with the hip joint being by far the most commonly affected joint, accounting for approximately 90% [7]. Clinical signs include swelling, hyperthermia with subsequent ossification of the joint-surrounding tissue and resulting limitation of motion [4, 8, 9].

There are already numerous publications on the histologic picture of HO, but these are either surgically resected mature HO or specimens from animal studies [10–12]. We succeeded in histologic examination of early-stage NHO from biopsies of nine spinal cord injured individuals. Thus we were not only able to show that a pathological change in the tissue begins very early and before the development of bone tissue, but that it also takes place in several stages. In addition, sonography has been confirmed as a reliable standard examination [13]. The distribution of NHO in the affected pelvic region was also further investigated.

## Material and methods

### Participants and controls

Nine participants with a complete spinal cord lesion grade A or B according to the American Spinal Injury Association Impairment Scale (AIS) as a component of the International Standards for Neurological Classification of SCI (ISNCSCI) and evidence of an acute NHO in the hip joint muscles were recruited for the study. In addition to obtaining a positive vote from the local ethics committee (Register No. 3712-10), an intervention-related patient insurance was granted, in case of a possible surgical complication, such as postoperative bleeding, infection, enhancement of NHO, and resulting costs for follow-up treatment. After detailed education and preparation, tissue was obtained and preserved from the hip joint tissue affected by NHO and from an unaffected calf, from the caput laterale of the M. gastrocnemius, as control by fine-needle biopsy under sonographic control. Both were subsequently examined histologically.

### NHO screening process and patient acquisition

As part of a screening procedure for early identification of the development of NHO, every 14 days between weeks 2 and 12 after spinal trauma, all newly injured patients underwent a sonographic examination of the hip joints and a routine laboratory examination, which included C-reactive protein (CRP) and leukocyte count. CRP and leukocyte count were selected because in a myositic phase, before the start of mineralization/ossification, an inflammatory reaction is more likely to be observed and at this time, for example, the bone-specific alkaline phosphatase is generally not yet altered. At 12 weeks after trauma, if there was a clinical suspicion of acute NHO with redness and hyperthermia of the affected soft tissues around the hip joint, a blood test and sonography of the surrounding tissue of the joints were also performed as above. All participants had clinical symptoms of NHO for no longer than four weeks.

In case of sonographic evidence of edema and/or calcification in the tissues surrounding the hip joint, magnetic resonance imaging (MR) or computed tomography (CT) of the pelvis was performed if MRI was contraindicated for other medical reasons. MRI and CT were selected as diagnostic methods because they are good at visualizing acute neurogenic heterotopic ossification or ossifications that have already developed and are comparatively less time-consuming and invasive than, for example, a 3-phase nuclear bone scan. If a radiological diagnosis of incipient NHO was confirmed, individuals were screened for inclusion and exclusion criteria for the study, after a detailed explanation.

### Inclusion and exclusion criteria

The inclusion criteria included a minimum age of 18 years, in addition to the participant’s written consent to the study. The neurological level of injury had to be at least at the level of dermatome TH12 or higher so that the puncture could not produce pain in the participants. Gender was not a factor. It did not matter whether the NHO involved the left, right, or both hip joints.

Exclusion and discontinuation criteria included a refusal to perform the study at any time on the part of the participant as well as an increased bleeding tendency at the time of puncture or skin irritations in the body area to be punctured. Furthermore, ventilated individuals in whom an uncontrollable respiratory insufficiency could not be excluded before or during the puncture were excluded. Due to the requirements of the ethics committee, we were allowed a maximum of 10 punctures.

### Sample collection

Fine needle punction was performed using a BARD Magnum® biopsy system (Karlsruhe, Germany). The correct puncture site was found sonographically using a 5 MHz linear transducer. A 16 G (1.6 mm) × 25 cm punch needle was used for the involved hip musculature tissue puncture and a 16 G (1.6 mm) × 10 cm punch needle was used for the calf musculature puncture. The study participant was positioned on his contralateral side for this purpose. The gluteal and lower leg region to be punctured was washed several times with skin disinfectant (Softasept® N) and covered sterilely. A linear transducer was used to locate the region of the NHO, which appeared hypersonor due to edema formation and, in the case of incipient ossification, with partly calcareous inclusions. After finding the region to be punctured, the skin in the area of the puncture was incised over a length of approx. 0.3 cm with a puncture scalpel (No. 11) down to the subcutis. The puncture needle was then advanced through this puncture site to the area of the NHO for specimen collection under sonographic control. Finally, a sterile dressing was applied. On the day following the puncture, the puncture areas affected by NHO were subjected to irradiation with 7 Gy (J/kg) and 15 MeV photons to prevent further ossification. Furthermore, the irradiation served to prevent a possible increase in NHO activity due to the puncture itself. However, radiation was not part of the study and was performed as standard in the presence of NHO regardless of the puncture.

Puncture of the lower leg was performed in the middle third of the gastrocnemius muscle. For this purpose, the shorter puncture needles as above were used. One of each of the thus obtained, approx. 1 cm long tissue strips from the gluteal and calf region was placed in a sample tube filled with formaldehyde and glutaraldehyde and thus preserved for further histological examination.

### Histological examination

Biopsy specimens from affected and unaffected body regions were processed for light microscopic examination using hematoxylin-eosin (HE) and elastica-van Gieson (EvG) staining.

## Results

All examined individuals showed a sensorimotor complete, grade AIS-A paraplegia according to the ISNSCI classification. In two patients the symptoms were present from dermatome level cervical C5 and in one case each cervical C1, C4 and C7 and thoracic Th6, Th7, Th9 and Th10. A total of seven men and two women with a mean age of 45.9 (SD 17.9, range 21–76) were examined.

Laboratory but nonspecific screening parameters included CRP and leukocyte count. Furthermore, sonography and CT or MRI imaging were performed just before the puncture biopsy to verify the onset of NHO. The participants’ puncture biopsies from the affected and unaffected tissues were examined by microscopy.

### Laboratory chemical examination of the blood

Routine blood examination showed no elevation of CRP in two participants, mild elevation with values between 1–10 mg/dl in five, and moderate elevations between 10–20 mg/dl in two (laboratory standard value for CRP ˂ 1.0 mg/dl). Except for a mild leukocytopenia of 3.3 in 1/nl in one case, normal values of the leukocyte count were detected in all other samples (Table 1).

**Table 1:** Routine laboratory test results.

Participants	CRP in mg/dl	Leukocyte count in 1/nl	Sonographic examination	MRI/CT	Histological examination
Normal value	<1.0	4–11			
POA/01	1.7	6.8	echoinhomogeneous oedema	oedema on both sides, incipient calcification on the right	oedema, fibrosis, haemorrhage
POA/02	13.7	9.6	echoinhomogeneous oedema	75 mm right gluteal ossification	necrosis, oedema, fibrosis, haemorrhage
POA/03	5.9	7	echoinhomogeneous oedema	oedema on both sides	oedema, fibrosis
POA/04	0.4	4.4	oedema on both sides, right gluteal calcifications	advanced ossification of the deep hip muscles	necrosis, oedema, fibrosis, calcification
POA/05	8.8	6	echoinhomogeneous oedema	oedema on both sides, accentuated on the right	oedema, fibrosis
POA/06	0.2	3.3	echoinhomogeneous oedema	right gluteal calcification onset	oedema, fibrosis
POA/07	2.7	7.9	echo-rich oedema	oedema on both sides, gluteal	oedema, fibrosis, haemorrhage, lipocytes
POA/08	10.6	7.2	echoinhomogeneous oedema	left gluteal 18 mm calcification	Necrosis, oedema, fibrosis
POA/09	7.2	9.3	echoinhomogeneous oedema	generalised oedema, incipient calcifications on both sides	oedema, fibrosis, haemorrhage, lipocytes

### Radiological examination

**Fig. 1:**
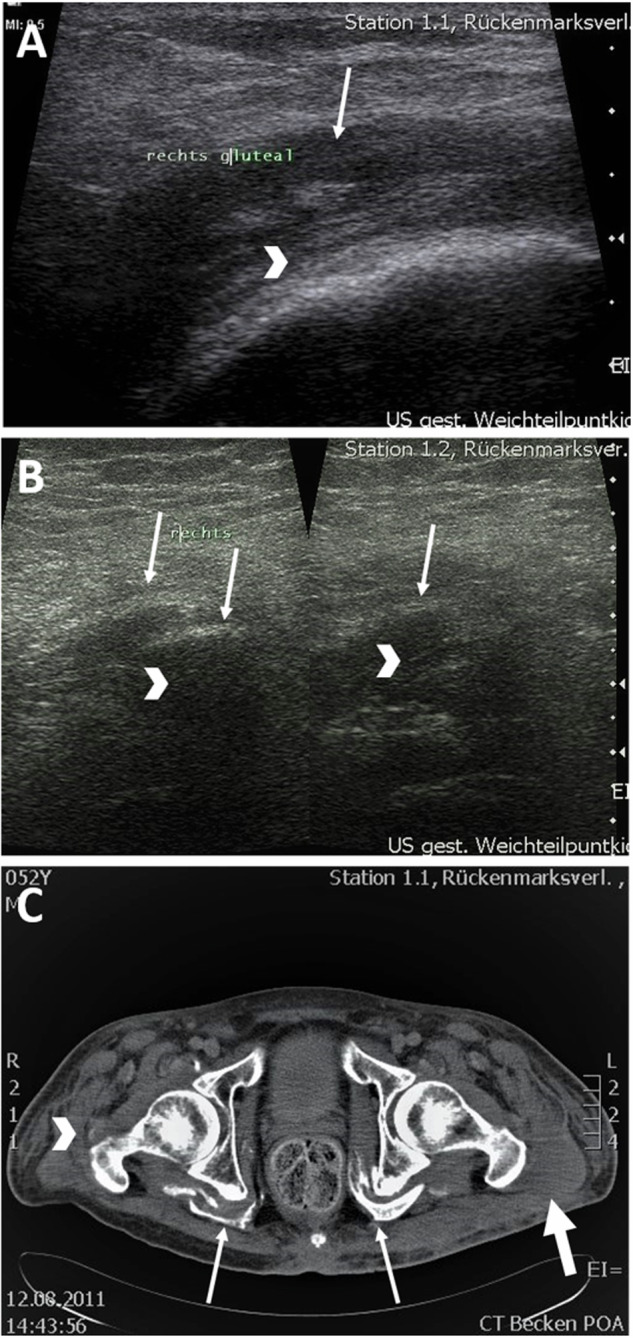
Sonographic images and computed tomography. **A** Sonographic image. Participant 4: edematous altered gluteal muscles (arrow) in the area of the greater trochanter (arrowhead). **B** Sonographic image. Participant 4: Calcifications (arrow) with dorsal sound extinction (arrowhead). **C** Computed tomography of the pelvis. Marked NHO in the area of the deep hip muscles (arrow) and incipient NHO in the area of the trochantor major as well as M. obturatorius on the right (arrowhead). Muscular edema (thick arrow).

In all sonographic examinations of the tissue surrounding the hip joint, pronounced edematous changes of the gluteal muscles could be detected (Fig. 1A). Leading in this regard were the M. gluteus medius, M. gluteus minimus, and to a lesser extent the M. gluteus maximus. Initial edema of the M. iliopsoas, M. obturatorius externus and internus as well as of the Mm. gemelli and the adductor muscles could also be detected. Bursitis trochanterica and/or iliopsotica was seen in four participants. In one participant, calcifications were detected sonographically (Fig. 1B).

MRI was performed in five participants for extended radiological diagnosis of NHO and CT in the remaining four participants. Here, congruent with the sonographic examination, gluteal edema of the muscles surrounding the hip joint could be seen. In six participants ossification could already be detected (Fig. 1C). Leadingly affected were, in descending order, the Mm. glutei medii, minimi, followed by Mm. glutei maximi, Mm. obturatorii, gemelli and iliopsoai. In one case, isolated calcification of the Mm. obturatorii, Mm. gemelli, and Mm. iliopsoai was demonstrated without involvement of the gluteal muscles. In two participants, no calcification was demonstrated in addition to generalized edema.

### Light microscopic examination of the specimens

**Fig. 2:**
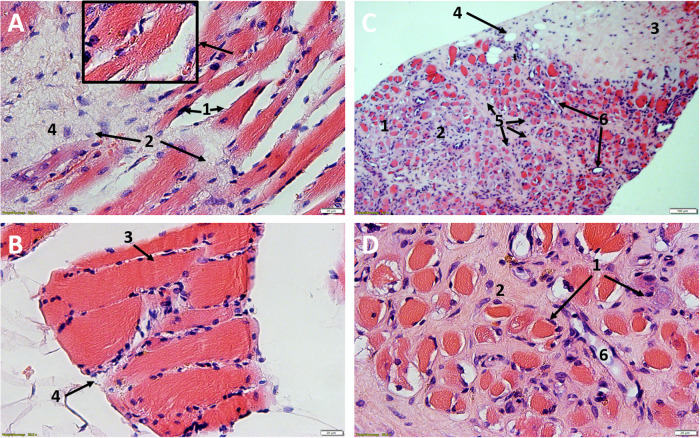
Light microscopic images. **A**/**B** Participant 2: HE, 25X magnification, Comparative images of the affected tissue **A** show atrophy of the myocytes with cell separation (1) and incipient fibrotic remodeling of the muscle (2). In contrast, healthy muscle **B** from the gastrocnemius muscle of the same participant. Easily recognizable marginal muscle cell nuclei (3) surrounded by endomysium and fibroblasts (4). Typical transverse striation of skeletal muscle (small box). **C**/**D** Participant 1: NHO, **C** HE, 6X magnification, **D** HE, 25X magnification. Advanced muscle atrophy and necrosis (1) with fibrotic destruction (2). Collagenous connective tissue with focal hemorrhage (3) and embedded univacuolar adipose tissue (4). Perimysium (5) with fibroblasts located within. Partially transversely and longitudinally sectioned vessels (6).

**Fig. 3:**
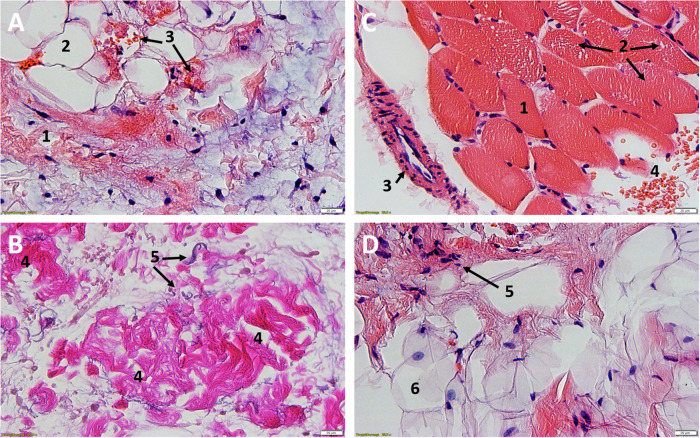
Light microscopic images. **A**/**B** Participant 9, NHO, **A** HE, 25X, **B** EvG, 25X. Complete fibrotic destruction with embedded fibroblasts (1), univacuolar adipose tissue (2), focal hemorrhage with dark orange stained erythrocytes (3). In the lower figure **B**, by EvG staining, the collagenous (4) fibers, as the main component of fibrosis, and the elastic (5) fibers can be shown separately. **C**/**D** Participant 7, **C** tissue from calf HE, 25X, **D** NHO, HE, 25X. Comparative healthy muscle with marginal nuclei (1), fixation artifact with myofibrils pushed apart (Cohnheim’s field) (2), arteriole (3), erythrocytes (4). Complete fibrotic and fat destruction **D**, fibroblasts (5) and univacuolar fat cells (6).

In every histological specimen from the NHO-affected gluteal region varying degrees of tissue deformation were detectable. The biopsies from the NHO affected gluteal region all showed varying degrees of fibrosis, stromal edema, muscle necrosis, adipose tissue in the form of univacuolar cells in varying degrees, and hemorrhage (Figs. 2 and 3). Edema was detectable in all samples resulting in divergent muscle fibres through the samples. In addition, all samples showed the beginning of fibrosis, which was already very clearly detectable in some cases. In these cases the muscle fibers often showed atrophy. These changes were sometimes detectable side by side, with individual samples showing necrosis and hemorrhages additionally.

The unaffected reference samples of all participants from the calf region showed regular muscular structure microscopically. Muscle fibers histologically cut longitudinally and transversely showed no terms of caliber fluctuations or myopathic changes. No inflammatory changes were detectable. Partially cross-sectional and/or longitudinally impacted muscle tissue with marginal cell nuclei and fixation-related fielding (Cohnheim’s field), marginal cell nuclei and surrounding peri- and endomysium could be detected (Figs. 2A/B and 3C/D).

## Discussion

**Fig. 4:**
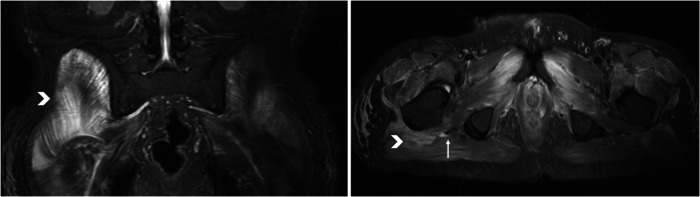
Embedded sciatic nerve (arrow) surrounded by muscular edema (arrowhead) on initial NHO on MRI of the pelvis (T2 sequence).

This study was the first to histologically examine puncture biopsies of early NHO. Histological studies of heterotopic ossifications in the hip joint region have been performed before, but these were intraoperative resections of fully mature HO and rarely after spinal cord injury [10–12]. The new finding here is, how tissue changes in the acute stage of NHO, and before calcification is developed. An altered muscle tissue pattern in comparison to healthy tissue was found in all participants. The tissue affected by NHO from the region of a hip joint showed atrophy and necrosis of the musculature in varying degrees in all participants. Leukocytosis infiltration could also be detected in several specimens, corresponding to a local inflammatory reaction. All specimens exhibited partly noticeable fibrotic destruction of the muscular tissue with adipose tissue remodeling. This correlated with the results of radiological diagnosis. Here, the gluteal muscles were most frequently affected by NHO, followed by the adductor and flexor muscle groups, confirming the results of previous studies [14]. Interestingly, a proximity of NHO to nerve pathways can be detected with striking frequency on computed tomography and MRI. Figure 4 shows the embedded sciatic nerve (arrow) surrounded by muscular edema (arrowhead) on initial NHO on MRI of the pelvis (T2 sequence). This may provide a bridge to studies suggesting a role for peripheral nerves in the development of NHO [15–17]. An influence of the peripheral nerves on bone healing after a fracture, with excessive callus formation, has also already been demonstrated [18, 19]. It is known that radiation therapy inhibits further differentiation of pluripotent cells and osteoblasts [20]. The exact affect this has on further histological differentiation and whether the process is even reversible remains a subject for further studies.

Although no work using human puncture biopsy material can be found in the literature, there are comparative animal studies and also studies using human tissue obtained intraoperatively [10–12]. Despite the difference that the individuals studied were not paraplegic and the samples were obtained intraoperatively rather than by biopsy, there is substantial overlap. All describe atrophic, necrotic, fatty, and fibrotic changes in the tissue studied.

**Fig. 5:**
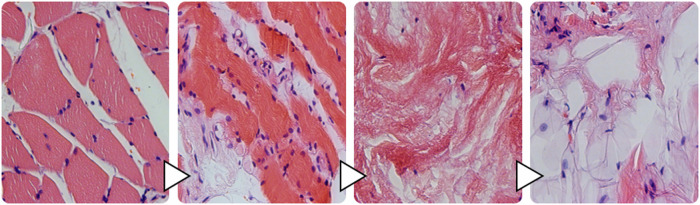
Cascade of NHO before enchondral ossification.

Considering a division of NHO into three phases according to Kaplan et al., the “early” NHO can also hypothetically be divided into several phases based on the present histological results [21]. After an initial local inflammation with leukocytic infiltration, there is local multi-perfusion, with edema and hemorrhage. Following muscle atrophy and necrosis, there is increased fibrotic and lipocytic remodeling of the affected tissue. Finally, cartilage tissue can be detected as the beginning of enchondral ossification (Fig. 5).

### Limitations

Although the studies of other research groups support the results of the present work, it is important to point out possible weaknesses of these studies. Varying results could have occurred due to the minimal invasiveness of sampling by biopsy. Six participants already had calcifications on MRI or CT, so despite the acute symptoms, it cannot be safely ruled out that there was not an advanced stage of NHO or possibly an “acute on chronic” condition. The time interval between the trauma and the puncture after the development of the NHO was on average 11.4 weeks (min. 3.43 weeks, max. 20.86 weeks, SD 5.58). Even if each biopsy was performed under sonographic control, there is still a possibility that, first, the center of NHO was not hit and, second, incipient ossification was not punctured because it was displaced or not captured by the tip of the puncture needle.

Even if the preparation of the tissue was performed in a certified laboratory, another source of error could be the preparation itself. In this case, cartilage and bone tissue could have been detached when cutting the tissue blocks for the slides and subsequently not displayed in the light microscope.

## Conclusions

The present work shows the tissue changes of a NHO after a spinal cord trauma from the first symptoms to the beginning of ossification. The difference to the previous studies lies in the way of sample collection by a puncture biopsy and not as before, the examination of resected tissue of the mature NHO. Sonographic examination is a highly regarded method of detecting NHO, but it must be assumed that at the first symptoms of NHO most participants already have ossifications of varying severity. Due to the strikingly frequent proximity of NHO to the peripheral nerve tracts in CT and MRI, the importance of the peripheral nerves in the development of NHO should be further investigated in additional radiological as well as clinical and experimental studies.

## Data Availability

All data generated or analyzed during this study are included in this published article [and its supplementary information files].
